# Supplementary material for: Chromosome and Genome Divergence between the Cryptic Eurasian Malaria Vector-Species Anopheles messeae and Anopheles daciae
Source: Genes (Basel). 2020 Feb 5;11(2):165. doi: 10.3390/genes11020165 (PMC7074279; doi:10.3390/genes11020165)
Supplement: Supplementary file 1 [file genes-11-00165-s001.pdf]

Supplementary table S1. ITS2 genotypes and inversion karyotypes in three populations of *An. messeae* in Moscow region. Letters in sample numbers indicate region (M – Moscow) and location (E – Yegoryevsk, N – Novokosino, No – Noginsk). The presence of double picks in ITS2 sequences is indicated by the IUPAC nucleotide ambiguity code: M – A and C, W – A and T, Y – T and C, R – A and G, S – C and G. Small letters indicate manually assigning base. X, 2R, 3R and 3L stand for chromosomal arms. Numbers in karyotypes represent standard (0) or inverted (1, 4) arrangements. Males can be recognized by a single arrangement number in X chromosome karyotype that corresponds to one homolog of this chromosome in males.

Sample number	Accession number	Species	SNP						Karyotype			
			150	211	215	217	412	432	X	2R	3R	3L
MN1	MG808170	*An. messeae*	M	T	T	C	G	G	1	00	01	00
MN2	MG808171	*An. messeae*	M	T	T	C	G	G	11	01	01	00
MN3	MG808172	*An. messeae*	M	T	T	C	G	G	11	01	00	00
MN4	MG808173	*An. daciae*	C	W	W	Y	A	C	01	00	01	00
MN5	MG808174	*An. daciae*	C	W	W	T	A	C	11	00	00	00
MN6	MG808175	*An. messeae*	M	T	T	C	G	G	1	01	01	00
MN7	MG808176	*An. messeae*	M	T	T	C	G	G	1	01	00	00
MN8	MG808177	*An. messeae*	M	T	T	C	G	G	14	00	01	01
MN9	MG808178	*An. messeae*	M	T	T	C	G	G	14	00	00	00
MN10	MG808179	*An. messeae*	M	T	T	C	G	G	11	00	00	00
MN11	MG808180	*An. messeae*	M	T	T	C	G	G	11	00	01	00
MN12	MG808181	*An. messeae*	M	T	T	C	G	G	1	00	00	00
MN13	MG808182	*An. messeae*	M	T	T	C	G	G	11	00	11	00
MN14	MG808183	*An. messeae*	M	T	T	C	G	G	11	00	01	01
MN15	MG808184	*An. messeae*	M	T	T	C	G	G	11	11	00	00
MN16	MG808185	*An. messeae*	M	T	T	C	G	G	11	11	01	00
MN17	MG808186	*An. daciae*	C	W	W	Y	A	C	0	00	00	00
MN19	MG808187	*An. messeae*	M	T	T	C	G	G	11	00	00	00
MN20	MG808188	*An. messeae*	M	T	T	C	G	G	1	00	00	00
MN21	MG808189	*An. messeae*	M	T	T	C	G	G	11	11	01	00
MN22	MG808190	*An. daciae*	C	W	W	Y	A	C	01	00	01	00
MN23	MG808191	*An. messeae*	M	T	T	C	G	G	11	01	00	00
MN24	MG808192	*An. messeae*	M	T	T	C	G	G	1	00	01	00
MN25	MG808193	*An. daciae*	C	W	W	Y	A	C	00	00	00	00
MN26	MG808194	*An. messeae*	M	T	T	C	G	G	11	11	01	00
MN27	MG808195	*An. messeae*	M	T	T	C	G	G	11	00	01	00
MN28	MG808196	*An. messeae*	M	T	T	C	G	G	11	01	00	00
MN29	MG808197	*An. messeae*	M	T	T	C	G	G	1	00	01	00
MN30	MG808198	*An. messeae*	m	T	T	C	G	G	11	01	01	00
MN31	MG808199	*An. messeae*	M	T	T	C	G	G	11	00	01	00
MN32	MG808200	*An. messeae*	M	T	T	C	G	G	1	01	01	00

MN33	MG808201	*An. messeae*	M	T	T	C	G	G	1	00	00	00
MN34	MG808202	*An. messeae*	M	T	T	C	G	G	11	00	00	00
MN35	MG808203	*An. daciae*	C	W	W	T	A	C	01	00	00	00
MN36	MG808204	*An. messeae*	M	T	T	C	G	G	1	00	00	00
MN37	MG808205	*An. messeae*	M	T	T	C	G	G	11	01	00	00
MN38	MG808206	*An. messeae*	M	T	T	C	G	G	11	01	00	00
MN39	MG808207	*An. messeae*	M	T	T	C	G	G	11	01	00	00
MN40	MG808208	*An. messeae*	M	T	T	C	G	G	11	00	00	00
MN41	MG808209	*An. messeae*	M	T	T	C	G	G	11	00	00	00
MN42	MG808210	*An. messeae*	M	T	T	C	G	G	11	00	01	00
MN43	MG808211	*An. messeae*	M	T	T	C	G	G	11	00	01	00
MN44	MG808212	*An. daciae*	C	W	W	Y	A	C	00	00	00	00
MN45	MG808213	*An. daciae*	C	W	W	Y	A	C	1	00	00	00
MN46	MG808214	*An. messeae*	M	T	T	C	G	G	11	01	00	01
MN47	MG808215	*An. daciae*	C	W	W	Y	A	C	1	00	01	00
MN48	MG808216	*An. messeae*	M	T	T	C	G	G	14	00	11	00
MN49	MG808217	*An. messeae*	M	T	T	C	G	G	11	01	00	00
MN50	MG808218	*An. messeae*	M	T	T	C	G	G	11	01	00	00
MN51	MG808219	*An. daciae*	C	W	W	Y	A	C	1	00	00	00
MN52	MG808220	*An. daciae*	C	W	W	Y	A	C	01	00	00	00
MN53	MG808221	*An. messeae*	M	T	T	C	G	G	11	01	00	00
MN54	MG808222	*An. messeae*	M	T	T	C	G	G	1	00	11	00
MN55	MG808223	*An. messeae*	M	T	T	C	G	G	1	01	00	01
MN56	MG808224	*An. messeae*	M	T	T	C	G	G	1	01	00	00
MN57	MG808225	*An. messeae*	M	T	T	C	G	G	11	01	00	00
MN58	MG808226	*An. daciae*	C	W	W	Y	A	C	0	00	00	00
MN59	MG808227	*An. messeae*	m	T	T	C	G	G	11	00	01	00
MN60	MG808228	*An. messeae*	M	T	T	C	G	G	11	00	00	00
MN61	MG808229	*An. messeae*	M	T	T	C	G	G	1	01	00	00
MN62	MG808230	*An. daciae*	C	W	W	Y	A	C	0	00	00	00
MN63	MG808231	*An. messeae*	M	T	T	C	G	G	11	00	00	00
MN64	MG808232	*An. messeae*	M	T	T	C	G	G	14	11	01	00
MN65	MG808233	*An. messeae*	M	T	T	C	G	G	11	00	01	00
MN66	MG808234	*An. daciae*	C	W	W	Y	A	C	01	00	00	00
MN67	MG808235	*An. messeae*	m	T	T	C	G	G	11	00	11	00
MN68	MG808236	*An. daciae*	C	W	W	Y	A	C	11	00	00	00
MN69	MG808237	*An. daciae*	c	W	W	y	A	C	0	00	00	00
MN70	MG808238	*An. messeae*	M	T	T	C	G	G	11	01	01	00
MN71	MG808239	*An. daciae*	C	W	W	y	A	C	01	00	00	00
MN72	MG808240	*An. messeae*	M	T	T	C	G	G	11	00	00	00
MN73	MG808241	*An. messeae*	M	T	T	C	G	G	1	11	00	00
MN74	MG808242	*An. messeae*	M	T	T	C	G	G	11	00	00	00

MN75	MG808243	*An. daciae*	C	W	W	Y	A	C	0	00	00	00
MN76	MG808244	*An. daciae*	C	W	W	Y	A	C	01	00	00	00
MN77	MG808245	*An. messeae*	M	T	T	C	G	G	14	00	01	00
MN78	MG808246	*An. messeae*	M	T	T	C	G	G	11	01	00	00
MN79	MG808247	*An. daciae*	C	W	W	Y	A	C	00	00	00	00
MN80	MG808248	*An. daciae*	C	W	W	Y	A	C	1	00	00	00
MN81	MG808249	*An. daciae*	C	W	W	Y	A	C	00	00	00	00
MN82	MG808250	*An. messeae*	M	T	T	C	G	G	1	00	00	00
MN83	MG808251	*An. messeae*	M	T	t	C	G	G	11	00	00	00
MN84	MG808252	*An. daciae*	C	W	W	Y	A	C	01	00	00	00
MN85	MG808253	*An. messeae*	m	T	T	C	G	G	1	01	00	00
MN86	MG808254	*An. daciae*	C	W	W	Y	A	C	11	00	01	00
MN87	MG808255	*An. messeae*	M	T	T	C	G	G	1	01	01	00
MN88	MG808256	*An. messeae*	M	T	T	C	G	G	11	00	00	00
MN89	MG808257	*An. messeae*	M	T	T	C	G	G	11	01	01	00
MN90	MG808258	*An. messeae*	M	T	T	C	G	G	11	11	01	00
MN91	MG808259	*An. messeae*	M	T	T	C	G	G	1	11	11	00
MN92	MG808260	*An. messeae*	M	T	T	C	G	G	14	00	01	00
MN93	MG808261	*An. daciae*	C	W	W	Y	A	C	00	00	00	00
MN94	MG808262	*An. messeae*	M	T	T	C	G	G	1	01	01	00
MN95	MG808263	*An. messeae*	M	T	T	C	G	G	14	11	00	00
MN96	MG808264	*An. messeae*	M	T	T	C	G	G	1	11	01	00
MN97	MG808265	*An. messeae*	M	T	T	C	G	G	14	00	01	00
MN98	MG808266	*An. messeae*	M	T	T	C	G	G	11	00	11	00
MN99	MG808267	*An. daciae*	C	W	W	Y	A	C	1	00	00	00
MN100	MG808268	*An. messeae*	M	T	T	C	G	G	11	00	11	00
MNo1	MG686138	*An. messeae*	M	T	T	C	G	G	1	11	00	00
MNo2	MG686139	*An. messeae*	m	T	T	C	G	G	11	00	01	00
MNo3	MG686140	*An. messeae*	m	T	T	C	G	G	11	01	00	01
MNo4	MG686141	*An. messeae*	M	T	T	C	G	G	1	00	00	00
MNo5	MG686142	*An. messeae*	M	T	T	C	G	G	11	00	01	00
MNo6	MG686143	*An. messeae*	M	T	T	C	G	G	1	01	01	00
MNo7	MG686144	*An. daciae*	C	W	W	Y	A	C	0	00	01	00
MNo8	MG686145	*An. daciae*	C	W	W	Y	A	C	00	00	00	00
MNo9	MG686146	*An. messeae*	M	T	T	C	G	G	11	01	01	00
MNo10	MG686147	*An. messeae*	M	T	T	C	G	G	11	01	01	00
MNo11	MG686148	*An. messeae*	M	T	T	C	G	G	1	11	00	00
MNo12	MG686149	*An. messeae*	M	T	T	C	G	G	4	11	00	00
MNo13	MG686150	*An. messeae*	M	T	T	C	G	G	1	01	01	00
MNo14	MG686151	*An. messeae*	M	T	T	C	G	G	11	01	11	00
MNo15	MG686152	*An. messeae*	M	T	T	C	G	G	11	11	00	01
MNo16	MG686153	*An. messeae*	M	T	T	C	G	G	1	00	01	00

MNo17	MG686154	*An. messeae*	M	T	T	C	G	G	1	11	00	00
MNo18	MG686155	*An. messeae*	m	T	T	C	G	G	14	00	00	00
MNo19	MG686156	*An. daciae*	C	W	W	Y	A	C	11	00	00	00
MNo20	MG686157	*An. messeae*	m	T	T	C	G	G	11	11	01	00
MNo21	MG686158	*An. daciae*	C	W	W	Y	A	C	11	00	00	00
MNo22	MG686159	*An. messeae*	M	T	T	C	G	G	11	11	00	00
MNo23	MG686160	*An. daciae*	C	W	W	Y	A	C	00	00	00	00
MNo24	MG686161	*An. messeae*	M	T	T	C	G	G	11	01	00	00
MNo25	MG686162	*An. messeae*	M	T	T	C	G	G	1	00	01	00
MNo26	MG686163	*An. daciae*	C	W	W	Y	A	C	00	00	00	00
MNo27	MG686164	*An. messeae*	M	T	T	C	G	G	11	01	01	01
MNo28	MG686165	*An. messeae*	M	T	T	C	G	G	1	01	01	00
MNo29	MG686166	*An. daciae*	C	W	W	Y	A	C	00	00	00	00
MNo30	MG686167	*An. messeae*	M	T	T	C	G	G	1	11	00	00
MNo31	MG686168	*An. messeae*	M	T	T	C	G	G	11	00	00	00
MNo32	MG686169	*An. messeae*	M	T	T	C	G	G	11	01	01	00
MNo33	MG686170	*An. daciae*	C	W	W	Y	A	C	11	00	00	00
MNo34	MG686171	*An. daciae*	C	W	W	Y	A	C	1	00	00	00
MNo35	MG686172	*An. messeae*	M	T	T	C	G	G	1	00	11	00
MNo36	MG686173	*An. daciae*	C	W	W	Y	A	C	01	00	00	00
MNo37	MG686174	*An. messeae*	M	T	T	C	G	G	1	00	00	00
MNo38	MG686175	*An. messeae*	M	T	T	C	G	G	1	11	00	00
MNo39	MG686176	*An. daciae*	C	W	W	Y	A	C	01	00	01	00
MNo40	MG686177	*An. messeae*	m	T	T	C	G	G	11	01	00	00
MNo41	MG686178	*An. daciae*	C	W	W	Y	A	C	01	00	00	00
MNo42	MG686179	*An. messeae*	M	T	T	C	G	G	11	11	00	00
MNo44	MG686181	*An. messeae*	M	T	T	C	G	G	11	01	01	00
MNo45	MG686182	*An. messeae*	M	T	T	C	G	G	1	01	01	00
MNo46	MG686183	*An. messeae*	M	T	T	C	G	G	1	00	01	00
MNo47	MG686184	*An. daciae*	C	W	W	Y	A	C	00	00	00	00
MNo48	MG686185	*An. messeae*	M	T	T	C	G	G	1	00	01	00
MNo49	MG686186	*An. messeae*	M	T	T	C	G	G	11	01	00	00
MNo50	MG686187	*An. daciae*	C	W	W	Y	A	C	01	00	00	00
MNo51	MG686188	*An. daciae*	C	W	W	Y	A	C	0	00	00	00
MNo52	MG686189	*An. daciae*	C	W	W	Y	A	C	0	00	01	00
MNo53	MG686190	*An. daciae*	C	W	W	Y	A	C	1	00	00	00
MNo54	MG686191	*An. daciae*	C	W	W	Y	A	C	0	00	01	00
MNo55	MG686192	*An. messeae*	M	T	T	C	G	G	11	00	00	01
MNo56	MG686193	*An. messeae*	M	T	T	C	G	G	1	00	01	00
MNo57	MG686194	*An. messeae*	M	T	T	C	G	G	1	11	01	00
MNo58	MG686195	*An. daciae*	C	W	W	Y	A	C	00	00	00	00
MNo60	MG686196	*An. messeae*	M	T	T	C	G	G	1	01	00	00

MNo61	MG686197	*An. messeae*	M	T	T	C	G	G	1	01	01	00
MNo62	MG686198	*An. messeae*	M	T	T	C	G	G	11	00	00	00
MNo64	MG686200	*An. messeae*	M	T	T	C	G	G	14	00	00	00
MNo65	MG686201	*An. messeae*	M	T	T	C	G	G	1	01	00	00
MNo66	MG686202	*An. messeae*	M	T	T	C	G	G	1	11	01	00
MNo67	MG686203	*An. daciae*	C	W	W	Y	A	C	01	00	00	00
MNo68	MG686204	*An. daciae*	C	W	W	Y	A	C	1	00	00	00
MNo69	MG686205	*An. messeae*	M	T	T	C	G	G	1	01	11	00
MNo71	MG686206	*An. messeae*	M	T	T	C	G	G	11	00	01	00
MNo72	MG686207	*An. messeae*	M	T	T	C	G	G	11	01	01	00
MNo73	MG686208	*An. messeae*	M	T	T	C	G	G	11	00	00	01
MNo74	MG686209	*An. messeae*	M	T	T	C	G	G	1	00	01	00
MNo76	MG686210	*An. messeae*	M	T	T	C	G	G	1	00	00	00
MNo77	MG686211	*An. messeae*	M	T	T	C	G	G	11	01	00	00
MNo78	MG686212	*An. daciae*	C	W	W	Y	A	C	01	00	00	00
MNo79	MG686213	*An. daciae*	C	W	W	Y	A	C	00	00	00	00
MNo80	MG686214	*An. messeae*	M	T	T	C	G	G	11	01	00	00
MNo81	MG686215	*An. messeae*	M	T	T	C	G	G	11	00	01	00
MNo82	MG686216	*An. daciae*	C	W	W	Y	A	C	1	00	00	00
MNo83	MG686217	*An. messeae*	M	T	T	C	G	G	11	00	01	00
MNo84	MG686218	*An. daciae*	C	W	W	Y	A	C	1	00	00	00
MNo85	MG686219	*An. daciae*	C	W	W	Y	A	C	1	00	00	00
MNo86	MG686220	*An. daciae*	C	W	W	Y	A	C	1	01	00	00
MNo87	MG686221	*An. messeae*	M	T	T	C	G	G	1	00	01	00
MNo88	MG686222	*An. daciae*	C	W	W	Y	A	C	01	00	00	00
MNo89	MG686223	*An. daciae*	C	W	W	Y	A	C	1	00	11	00
MNo90	MG686224	*An. messeae*	M	T	T	C	G	G	11	00	11	00
MNo92	MG686225	*An. messeae*	M	T	T	C	G	G	11	11	01	01
MNo93	MG686226	*An. messeae*	M	T	T	C	G	G	1	00	00	00
MNo94	MG686227	*An. messeae*	M	T	T	C	G	G	11	00	01	01
MNo95	MG686228	*An. messeae*	M	T	T	C	G	G	11	01	00	00
MNo96	MG686229	*An. messeae*	M	T	T	C	G	G	1	01	00	00
MNo97	MG686230	*An. daciae*	C	W	W	Y	A	C	0	00	00	00
MNo98	MG686231	*An. messeae*	M	T	T	C	G	G	1	01	00	00
MNo99	MG686232	*An. daciae*	C	W	W	Y	A	C	11	00	00	00
MNo100	MG686233	*An. messeae*	M	T	T	C	G	G	11	01	01	00
ME1	MG727761	*An. daciae*	M	W	W	Y	A	C	00	00	00	00
ME2	MG727762	*An. daciae*	C	W	W	Y	A	C	0	00	00	00
ME3	MG727763	*An. messeae*	M	T	T	C	G	G	11	11	00	00
ME4	MG727764	*An. daciae*	C	W	W	Y	A	C	11	00	00	00
ME6	MG727765	*An. daciae*	M	W	W	Y	A	C	11	00	00	00
ME7	MG727766	*An. daciae*	C	W	W	Y	A	C	0	00	00	00

ME10	MG727767	*An. daciae*	C	W	W	Y	A	C	01	00	00	00
ME11	MG727768	*An. daciae*	C	W	W	Y	A	C	01	00	00	00
ME12	MG727769	*An. daciae*	C	W	W	Y	A	C	01	00	00	00
ME13	MG727770	*An. messeae*	M	T	T	C	G	G	11	01	01	00
ME14	MG727771	*An. messeae*	M	T	T	C	G	G	1	01	00	01
ME15	MG727772	*An. daciae*	C	W	W	Y	A	C	01	00	00	00
ME16	MG727773	*An. messeae*	M	T	T	C	G	G	11	00	11	00
ME17	MG727774	*An. daciae*	C	W	W	Y	A	C	01	00	00	00
ME18	MG727775	*An. messeae*	M	T	T	C	G	G	11	00	00	00
ME19	MG727776	*An. daciae*	C	W	W	Y	A	C	00	00	00	00
ME20	MG727777	*An. messeae*	M	T	T	C	G	G	11	00	00	01
ME21	MG727778	Hybrid	M	W	W	Y	R	S	04	00	00	00
ME22	MG727779	*An. daciae*	C	W	W	Y	A	C	01	00	00	00
ME23	MG727780	*An. daciae*	C	W	W	Y	A	C	1	00	00	00
ME24	MG727781	*An. messeae*	M	T	T	C	G	G	11	01	01	00
ME25	MG727782	*An. messeae*	M	T	T	C	G	G	4	00	00	00
ME26	MG727783	*An. messeae*	M	T	T	C	G	G	11	00	00	00
ME27	MG727784	*An. daciae*	C	W	W	Y	A	C	0	00	00	00
ME28	MG727785	*An. daciae*	C	W	W	Y	A	C	0	00	00	00
ME29	MG727786	*An. messeae*	M	T	T	C	G	G	1	00	01	01
ME30	MG727787	*An. daciae*	C	W	W	Y	A	C	1	00	00	00
ME32	MG727788	*An. daciae*	C	W	W	Y	A	C	1	00	00	00
ME33	MG727789	*An. daciae*	C	W	W	Y	A	C	01	00	00	00
ME34	MG727790	*An. daciae*	C	W	W	Y	A	C	00	00	00	00
ME36	MG727791	*An. daciae*	C	W	W	Y	A	C	1	00	00	00
ME37	MG727792	*An. daciae*	C	W	W	Y	A	C	1	00	00	00
ME38	MG727793	*An. daciae*	C	W	W	Y	A	C	0	00	00	00
ME39	MG727794	*An. daciae*	C	W	W	Y	A	C	1	00	00	00
ME40	MG727795	*An. daciae*	C	W	W	Y	A	C	1	00	00	00
ME41	MG727796	*An. daciae*	C	W	W	Y	A	C	0	04*	00	00
ME42	MG727797	*An. daciae*	C	W	W	Y	A	C	01	00	00	00
ME43	MG727798	*An. daciae*	C	W	W	Y	A	C	01	00	00	00
ME44	MG727799	*An. daciae*	C	W	W	Y	A	C	11	00	00	00
ME45	MG727800	*An. daciae*	C	W	W	y	A	C	1	00	00	00
ME46	MG727801	*An. messeae*	M	T	T	C	G	G	1	00	00	01
ME47	MG727802	*An. daciae*	C	W	W	Y	A	C	0	00	00	00
ME49	MG727803	*An. daciae*	C	W	W	Y	A	C	00	00	00	00
ME50	MG727804	*An. daciae*	C	W	W	Y	A	C	11	00	00	00
ME51	MG727805	*An. daciae*	C	W	W	Y	A	C	01	00	00	00
ME52	MG727806	*An. daciae*	C	W	W	Y	A	C	1	00	00	00
ME53	MG727807	*An. daciae*	C	W	W	Y	A	C	0	00	01	00
ME55	MG727808	*An. daciae*	C	W	W	Y	A	C	0	00	01	00

ME56	MG727809	*An. daciae*	C	W	W	Y	A	C	1	00	00	00
ME57	MG727810	*An. daciae*	C	W	W	Y	A	C	01	00	00	00
ME58	MG727811	*An. daciae*	C	W	W	Y	A	C	11	01	00	00
ME61	MG727812	*An. messeae*	M	T	T	C	G	G	1	00	00	00
ME62	MG727813	*An. messeae*	M	T	T	C	G	G	11	01	00	00
ME63	MG727814	*An. daciae*	C	W	W	Y	A	C	00	00	00	00
ME64	MG727815	*An. messeae*	M	T	T	C	G	G	11	00	00	00
ME65	MG727816	*An. messeae*	M	T	T	C	G	G	11	01	01	00
ME66	MG727817	*An. daciae*	C	W	W	Y	A	C	1	00	00	00
ME67	MG727818	*An. daciae*	C	W	W	Y	A	C	1	00	00	00
ME68	MG727819	*An. daciae*	C	W	W	Y	A	C	00	00	00	00
ME69	MG727820	*An. messeae*	M	T	T	C	G	G	11	01	00	00
ME71	MG727821	*An. daciae*	C	W	W	Y	A	C	01	00	00	00
ME72	MG727822	*An. daciae*	C	W	W	Y	A	C	00	00	00	00
ME73	MG727823	*An. messeae*	M	T	T	C	G	G	1	00	00	00
ME74	MG727824	*An. daciae*	C	W	W	Y	A	C	00	00	00	00
ME75	MG727825	*An. daciae*	C	W	W	Y	A	C	01	00	00	00
ME76	MG727826	*An. messeae*	M	T	T	C	G	G	1	00	00	01
ME77	MG727827	*An. messeae*	M	T	T	C	G	G	11	00	00	00
ME78	MG727828	*An. daciae*	C	W	W	Y	A	C	0	00	01	00
ME79	MG727829	*An. daciae*	C	W	W	Y	A	C	01	00	00	00
ME80	MG727830	*An. daciae*	C	W	W	Y	A	C	1	00	01	00
ME81	MG727831	*An. daciae*	C	W	W	Y	A	C	01	00	00	00
ME82	MG727832	*An. messeae*	M	T	T	C	G	G	1	00	00	00
ME83	MG727833	*An. messeae*	M	T	T	C	G	G	11	00	00	00
ME84	MG727834	*An. daciae*	C	W	W	Y	A	C	0	00	01	00
ME85	MG727835	*An. daciae*	C	W	W	Y	A	C	1	00	00	00
ME86	MG727836	*An. daciae*	C	W	W	Y	A	C	01	00	00	00
ME88	MG727837	*An. daciae*	C	W	W	Y	A	C	1	00	00	00
ME89	MG727838	*An. daciae*	C	W	W	Y	A	C	0	00	00	00
ME90	MG727839	*An. daciae*	C	W	W	y	A	C	00	00	00	00
ME91	MG727840	*An. daciae*	C	W	W	Y	A	C	11	00	00	00
ME92	MG727841	*An. messeae*	M	T	T	C	G	G	11	01	01	00
ME93	MG727842	*An. daciae*	C	W	W	Y	A	C	0	00	00	00
ME96	MG727843	*An. daciae*	C	W	W	Y	A	C	01	00	00	00
ME97	MG727844	*An. messeae*	M	T	T	C	G	G	11	00	01	00
ME98	MG727845	*An. daciae*	C	W	W	Y	A	C	0	00	00	00
ME99	MG727846	*An. messeae*	M	T	T	C	G	G	1	00	01	00
ME100	MG727847	*An. daciae*	C	W	W	Y	A	C	01	00	00	00

Supplementary table S2. Numbers of individuals with standard and inverted chromosomal arrangements in three Moscow populations of *An. messeae* and *An. daciae*. Populations are indicated as following: M – Moscow; E – Yegoryevsk: N – Novokosino; and No – Noginsk. X, 2R, 3R and 3L stand for chromosomal arms. Numbers in karyotype variants represent standard (0) or inverted (1, 4) arrangement.

Chromosome		Numbers of individuals in populations of different species					
Arm	Variant	MN		MNo		ME	
		An. messeae	*An. daciae*	*An. messeae*	*An. daciae*	*An. messeae*	*An. daciae*
X	0 & 00	0	10	0	12	0	23
	1 & 11	65	8	60	12	23	21
	01	0	8	0	7	0	18
	4	0	0	1	0	1	0
	14	8	0	2	0	0	0
2R	0 & 00	38	26	25	30	16	60
	1 & 11	10	0	13	0	1	0
	01	25	0	25	1	7	1
	04	0	0	0	0	0	1
3R	0 & 00	37	22	30	26	16	57
	1 & 11	7	0	4	1	1	0
	01	29	4	29	4	7	5
3L	0 & 00	69	26	56	31	19	62
	1 & 11	0	0	0	0	0	0
	01	4	0	7	0	5	0
